# White Blood Cell Count and Serum Cytokine Profile in Tropical Hardwood Workers in Kumasi

**DOI:** 10.1155/2022/8245717

**Published:** 2022-06-27

**Authors:** Isaac Ekow Ennin, Margaret Agyei Frempong, Daniel Dodoo, Francis A. Yeboah, Raymond Saa-Eru Maalman

**Affiliations:** ^1^Department of Basic Medical Sciences, School of Medicine, University of Health and Allied Sciences, Ho, Volta Region, Ghana; ^2^Kwame Nkrumah University of Science and Technology, Kumasi, Ashanti Region, Ghana; ^3^University of Ghana, Legon, Greater Accra, Ghana

## Abstract

**Background:**

Occupational exposure to wood dust particles has long been reported of its associated varying degrees of negative health effects due to different extractive chemicals present in the various timber species. However, tropical hardwood is also reported to have higher levels of extractive chemicals of antihistamine, antioxidant, and anti-inflammatory properties. In Ghana, woodworkers have for years been exposed to wood dust from mixed tropical hardwood species, with little or no protective equipment such as nose masks, yet with less significant respiratory conditions. This study seeks to investigate the serum cytokine profile in tropical hardwood workers in Kumasi to provide a better understanding of the immunoregulatory pattern activated in the woodworkers.

**Method:**

The study was carried out among woodworkers, teachers, and security men located in Kumasi. A cross-sectional sampling of adult male workers was selected to participate in the study (86 woodworkers and 89 nonwoodworkers). Participants donated blood collected by venepuncture into EDTA tubes and spun to separate serum for cytokine assay. Cytokines including IFN-gamma, IL-1*β*, IL-2, IL-4, IL-6, IL-10, IL-12, IL-13, and IL-17 were assayed using the Human Premixed Multianalyte Kit (R&D System, Inc., Minneapolis, USA) following the manufacturer's procedure. The cytokine levels were quantified using the Luminex∗200 analyser.

**Results:**

The mean concentration levels for the various cytokines were significantly different (*p* < 0.05) between woodworkers and nonwoodworkers except IL-2. There were significantly increased levels of Th1 and Th2 cytokines expressed in the woodworkers more than the nonwoodworkers.

**Conclusions:**

The results from this study reveal that exposed woodworkers of mixed tropical hardwood species show a high level of Th1 and Th2 cytokines in their serum than nonwoodworkers.

## 1. Introduction

Cytokines are a diverse group of peptides produced by different cell types including cancer cells and various subpopulations of immune system cells. They exert the effect as cellular mediators after binding to appropriate membrane receptors [1]. They are synthesized by both stationed and moving cells including mast cells, macrophages, lymphocytes, basophils, eosinophils, and neutrophils that have been activated [2]. Cytokines participate in different physiological and pathological processes: including the regulation of complex immune responses to pathogens, as well as inflammatory responses as they regulate their duration and intensity [1]. Cytokine plays the role of balancing communications between diverse cells in complex immune system responses, by transmitting signals from one cell type to another; necessary for cell activation, cell suppression, and a regulatory function [1].

Several classifications of cytokines have been proposed. The simple classification used by most authors are based on the cell type that produces the individual cytokines [1]. Other classifications group cytokines based on the potential to stimulate cells of the immune system, also classified as Th1 cytokines (IL-2, IL-12, IL-18, IL-21, IL-23, and IFN-*γ*); cytokines with inhibitory effects on the cell immune system also classified as anti-inflammatory cytokines (TNF-*α* and IL-10); cytokines that effect the regulatory balance of cells of the immune system (IL-10 and TGF-*β*); Th2 cytokines are classic mediators of the immune response which recognize extracellular parasite and promote IgE and eosinophilic response in atopy (IL-4, IL-5, IL-10, and IL-13) [1, 3, 4]; proinflammatory cytokines stimulate inflammation and play a significant role in immune-oncology [1].

Infiltration of inflammatory cells such as mast cells, lymphocytes, eosinophils, neutrophils, basophils, and macrophages to the site of inflammation has been identified in inflammatory diseases such as allergic rhinitis asthma and bronchitis in temperate woodworkers [5, 6]. Each of these cell types has a distinct role in the immune system and communicates with other immune cells using secreted cytokines [7]. Some temperate softwood and hardwood studied have shown induction or suppression of major proinflammatory cytokines (TNF-*α*, IL-1B, and IL-6) and CD4+effector subset cytokines Th1 (IFN-*γ*, IL-12) and Th2 (IL-4, IL-13), Treg (TGF-*β*, IL-10) cytokines, and chemokines (CCL3 CCL8, CCL11, CCL17, and E-selectin) in mouse alveolar macrophage cells by the wood dust [8–10]. Tropical hardwood species have been shown to have high levels of useful extractive compounds such as flavonoids which present significant anti-inflammatory effects causing suppression or inhibition of proinflammatory mediators [2, 7, 11, 12]. The serum levels of various cytokines may give information on the presence or even predictive value of inflammatory processes [13] occurring in the woodworker. The current study investigated the serum cytokine levels induced in tropical hardwood woodworkers in Kumasi.

## 2. Methods

### 2.1. Characteristics of Study Populations

This was a cross-sectional study to investigate the cytokine levels induced in tropical hardwood workers compared with nonexposed healthy control. A total of 86 adult male woodworkers were selected by cluster and convenient sampling from Sokoban Wood Village and recruited for the study. The mean age of the woodworkers was 38.55 ± 11.12 years with a body temperature range of 34.4–37.3°C. None of the woodworkers was on any medication within the period of study. A control group of 89 adult male teachers and security staff from the Kwame Nkrumah University of Science and Technology (KNUST) Basic School and KNUST Security Services who had no history of exposure to industrial air pollutants were selected for the study. The mean age of the control group was 42.74 ± 9.43 years with a body temperature range of 34.7–37.5°C.

### 2.2. Blood Sample Collection

About 10 ml of blood was collected from each participant by venepuncture using sterile disposable hypodermal syringes and needles. The blood was decanted 5 ml each into two vacutainer tubes: a Becton Dickinson Vacutainer Hemogard serum separator tube and an ethylene diamine tetra-acetic acid (EDTA) tube. Aliquots from the EDTA tubes were taken for blood cell count. In addition, serum samples were obtained from the serum separator tubes by centrifugation at 800×g for 5 minutes at the KNUST Hospital Medical Laboratory and stored at -80°C at Kumasi Centre Collaborative Research (KCCR), KNUST, until used for cytokine analysis utilizing immunoassays.

### 2.3. Blood Cell Count

The white blood cells and platelet count were determined using the Sysmex XP-300™ Automated Haematology analyser and the results printed on thermal paper. Procedures were followed as instructed in the operational manual.

### 2.4. Cytokine Analysis

Serum cytokines were analysed by the multiplex ELISA technique using the Luminex Human Premixed Multianalyte Kit (R&D System, Inc., Minneapolis, USA) according to the manufacturer's instructions. The kit detected all the 9 cytokines (IFN-*γ*, IL-1*β*, IL-2, IL-4, IL-6, IL-10, IL-12, IL-13, and IL-17) simultaneously in a single sample. Each kit contained Human Standard Cocktail, Human Premixed Microparticle Cocktail, Human Premixed Biotin-Ab Cocktail, Streptavidin–Phytoerythrin (PE) Concentrate, Diluent, Calibrator Diluent, Wash Buffer Concentrate, 96-well Microplate, Mixing Bottles Plate Sealer, and a Certificate of Analysis.

All the reagents and samples were brought to room temperature (RT) and an assay was performed at RT. The 96-well filter-bottomed microplate was prewet by filling each well with 100 *μ*l of wash buffer and removed using a vacuum manifold designed to accommodate a microplate. In each well, 50 *μ*l of a diluted cocktail of microparticles was added to the prewet filtered bottom microplate. In addition, 50 *μ*l of the standard cocktail was added to the first 6 wells, while to each of the rest of the wells, 50 *μ*l of each sample was added and all securely covered with a foil plate. It was then incubated for 2 hours at room temperature on the horizontal orbital microplate shaker set at 500 rpm. Using the vacuum manifold device, the microplate was washed by first removing the liquid, refilling each well with 100 *μ*l of wash buffer, and again removing the liquid. The washing procedure was repeated 3 times. After washing, 50 *μ*l of diluted biotin antibody cocktail was added to each well, and the plate was securely covered with a new foil plate sealer and incubated for 1 hour at room temperature on the shaker set at 500 rpm. Again, the liquid was removed, and the washing procedure was repeated three times as described above. After washing, 50 *μ*l of diluted Streptavidin-PE was added to each well, securely covered with a new foil plate sealer, and incubated for 30 minutes at room temperature on the shaker set at 500 rpm.

The liquid was removed, and washing was repeated three times as before. The microparticles were resuspended by adding 100 *μ*l of wash buffer to each well. The microplate was incubated for 2 minutes at room temperature on the shaker set at 500 rmp. It was read using the Luminex∗200 (manufacturer: Luminex Corporation, United States of America) analyser within 30 minutes. The procedure was used to measure cytokine levels for all the samples. Cytokine concentrations were determined from standard curves prepared on each plate and expressed in picogram per millilitre (pg/ml).

### 2.5. Statistical Analysis

At the end of the study, the field data was entered directly into Microsoft Excel 2010 statistical package and analysed using STATA version 14. The level of statistical significance was set at a *p* value < 0.05. Statistical analysis was conducted using Student's *t*-test to compare the inflammatory cell counts and the plasma cytokine levels between the exposed woodworkers and nonexposed workers. Spearman rank correlation was used to determine the correlation between the cytokines and the ages of woodworkers. Also, a correlation between the white blood cells and the cytokines was made. All continuous variables were expressed as means ± standard deviation.

## 3. Result

### 3.1. Comparison of Inflammatory Cell Counts between Study Populations

The total white blood cells, as well as the differential counts of lymphocytes, neutrophils, and platelets, were compared between the woodworkers and the nonexposed workers using Student's *t*-test. The total white blood cells, neutrophils, and platelets count were significantly higher in the woodworkers than the nonexposed workers, even though the total white blood cells were only marginally significant. However, the difference in the lymphocyte counts between the woodworkers and the nonexposed workers was not significant (Table 1).

### 3.2. Comparison of Cytokine Levels Expressed in Woodworkers with Nonexposed Workers

The level of cytokines expressed in the woodworkers and nonexposed workers is shown in Table 2. The Student's *t*-test was used to compare the mean levels of the various cytokines studied at (*p* < 0.05). Except for IL-2 which was insignificantly higher, all the other cytokines (IL-1*β*, IL-4, IL-6, IL-10, IL-12, IL-13, IL-17, and IFN-y) studied were significantly higher in the woodworkers than the nonexposed workers.

### 3.3. Determining the Classes of Cytokines Measured

The classes of cytokines measured with a significant difference in the woodworkers are shown in italics in Table 2. The major proinflammatory cytokine (IL-1*β*) and major CD4+ effect subsets cytokines: Th1 (IFN-y and IL-12p70), Th2 (IL-4 and IL-13) Treg (IL-10), and Th17 (IL-17) cytokines were all significantly higher in the woodworkers.

### 3.4. Correlation between Cytokines and Ages of Woodworkers

In investigating the correlations between cytokines and the ages of woodworkers in years, Spearman rank correlation was used at 5% significance level.

Table 3 shows that although there is a weak correlation between the various cytokines and the ages of woodworkers, none of the relationships is statistically significant (*p* > 0.05).

### 3.5. Ethical Issue

The study was carried out in accordance with the guidelines and principles of the Helsinki's Declaration of research involving human participants. The basic ethical principles such as respect for persons, beneficence, and justice were adhered to in the performance of the study. The study received ethical approval from the Committee on Human Research, Publication and Ethics (CHRPE) of the School of Medical Sciences of KNUST and Komfo Anokye Teaching Hospital (KATH) Kumasi (Ref: CHRPE/AP/304/15). A written request indicating the purpose and benefits of the research was sent to the leadership of each of the participating group to seek their permission to conduct research among their members. A durbar was held at each of the sites during which the purpose and benefits of the research was explained to them in English and the Ghanaian Language (Twi). All the participants completed informed consent form and received date to return for commencement of study. The participation in the study was voluntary, and respondents could withdraw at any stage of the data collection process. The anonymity of participants was ensured by using codes for individual answered questionnaire received.

## 4. Discussion

### 4.1. Comparison of Inflammatory Cell Counts

Increased inflammatory cell and platelet count in the peripheral blood is an indication of inflammatory reaction in the host. In the present study, the total white blood cell, neutrophil, and platelet counts were higher in the woodworkers. This is consistent with the result of Gripenbäck et al. [14] who found a significantly increased number of neutrophils in the peripheral blood of healthy volunteers who were exposed to pinewood dust. The lymphocyte count was not significantly high in the woodworkers. This result is consistent with the findings of Gripenbäck et al. [14] who found significant decreased lymphocyte numbers in the peripheral blood of healthy volunteers exposed to wood dust and also found increased lymphocytes in the bronchoalveolar lavage (BAL) fluid. The increased inflammatory cells in the peripheral blood in this study suggest that the wood dust may have induced inflammatory reactions in the woodworkers.

### 4.2. Comparison of Cytokine Levels among Woodworkers and Nonexposed Workers

Cytokines are inflammatory and immunomodulating mediators that exhibit both negative and positive regulatory effects on various target cells [15]; hence, the serum levels of various cytokines may give information on the presence of inflammatory processes [13]. This study investigated the cytokine profile of the different classes of cytokines in the woodworkers.

Proinflammatory cytokines promote inflammation by activating a variety of proinflammatory genes, including phospholipase A2, cyclooxygenase 2 (COX-2), inducible nitric oxide synthase (iNOS), and other cytokines and chemokines to initiate inflammation [16–18]. IL-1*β* and IL-6 also promote the development of T helper (Th17) cells and Th1 cell lineage [19, 20]. The proinflammatory cytokines (IL-1*β* and IL-6) were increased in the woodworkers in this study, which suggests that the wood dust induces proinflammatory cytokines and therefore may promote inflammatory reaction in the woodworkers. This observation is similar to the result of Long et al. [9], Bornholdt et al. [8] and Määttä et al. [6] in which wood dust of all the tested wood species induced proinflammatory cytokine expression in the human lung tissue and animal model studies. The present study, therefore, supports the previous observation that the wood dust induces an inflammatory response in the woodworkers.

The CD4+ effector subset Th1, Th2, Th17, and Treg cytokines were higher in the woodworkers, which suggests that the wood dust may have induced Th1, Th2, Th17, and Treg immune response in the woodworkers. However, no significant changes have been reported in previous studies on the expression of Th1, Th2, Th17, and Treg cytokines in woodworkers, human tissue, or animals exposed to wood dust [10, 21]. Again, there were no studies reported on CD4+ effector subset cytokine induction in woodworkers exposed to tropical hardwood.

The Th1 cytokines (IFN-*γ* and IL-12) are known to mediate the killing of intracellular pathogens by activating monocytes and macrophages to increase their cytokine secretion and antigen presentation. However, they also resist the Th2 cell cytokine function and Th2 response (allergic response) [3, 4]. Th1 cytokines also downregulate eosinophil differentiation by suppressing the development of Th2 cells in allergic inflammation [22]. This study, therefore, suggests that the increased Th1 cytokine levels observed in the woodworkers may contribute to resisting allergic inflammatory reactions that may be induced due to the increase Th2 level in the woodworkers.

This is consistent with the findings that altering the cytokine-producing profile of Th2 cells by inducing Th1 responses is protective against Th2-related disorders such as asthma and allergy [23]. This study suggests that the Th1 response induced by the wood dust may contribute to resisting allergic response in woodworkers. This may have contributed to the normal lung function indices observed in the woodworkers in our study (not published).

The Th2 cytokines (IL-4 and IL-13) were higher in the woodworkers compared to the nonexposed workers, suggesting that the wood dust may have contributed to induce of Th2 response in the woodworkers. Th2 cytokines are important in hypersensitivity reactions and allergic immunopathology. They are known to enhance mucus release, class switching of B cells to produce IgE, and fibrosis [14]. The action of the Th2 immune response can result in IgE production, inflammation of the airways, and tissue remodelling [22]. Although IgE was not measured in the present study, the increased Th2 cytokines may support the high level of IgE found in a previous study of exposed tropical hardwood workers in Accra [24]. The increased Th2 cytokines in the woodworkers in the present study may have contributed to allergic respiratory symptoms such as sneezing and catarrh and mucous release woodworkers. This is consistent with the findings of Gripenbäck et al. [14] and Shum et al. [25] that elevated production of the Th2 cytokines contributes to allergic airway inflammation. The high levels of the Th1 cytokines (IFN-*γ* and IL-12) observed in the present study may be attributed to the high level of Th2 cytokines (IL-4) to antagonize the development of Th2 response and downregulate the Th2 inflammatory reactions in the woodworkers [4, 25].

The Th17 cytokine IL-17A was higher in the woodworkers suggesting that the wood dust may have induced a Th17 response in the woodworkers. IL-17A is known to stimulate the production of other proinflammatory cytokines and play protective roles in host resistance against pathogens at epithelial and mucosal barriers [26, 27]. Elevated levels of IL-17A have been observed in the airways of asthmatics and are associated with neutrophil influx [25]. In the present study, the IL-17A level was higher in the woodworkers, this indicating that IL-17A may contribute to the induction of inflammation reaction in the woodworkers.

The woodworkers had a higher Treg cytokine (IL-10) level than the nonexposed workers suggesting an increased Treg response in the woodworkers. Activated Treg produces IL-10 which inhibits the synthesis of proinflammatory cytokines or suppresses their activities, hence negatively modulating inflammatory response [28]. It also reduces airway hyperreactivity (AHR), lung eosinophil infiltration, and Th2 cytokine production which are characteristics of allergic airway inflammation. IL-10 together with IL-4 and IL-13 downregulates immunological response in the lungs [3, 4, 29, 30]. The higher level of Treg cytokine in the present study suggests that the increased Treg cytokine in the woodworkers may contribute to the suppression of allergic and inflammatory reactions induced by wood dust to limit the immunopathological occurrence in the woodworkers.

### 4.3. Correlation between Cytokines and Ages of Woodworkers

In previous studies, cytokines have been linked to the aging process, inflammatory cytokines such IL-1*β*, IL-6, and TNF-*α* have been recorded to increase with aging [31–33]. The present study shows a nonsignificant positive correlation of cytokine (IL-1*β*, IFN-*γ*, IL-4, IL-6, IL-13, IL-17A, and IL-12p70) levels with the age of the woodworkers and a nonsignificant negative correlation of cytokine (IL-10 and IL-2) levels with the age of the woodworkers. Could this be a result of the wood dust stimulating inflammatory suppressors in the woodworkers?

## 5. Conclusion

The present study reveals that mixed tropical hardwood dust may contribute to inducing allergic inflammatory responses in the woodworkers which are evident by the elevated inflammatory cells and proinflammatory and Th2 cytokines. Moreover, the wood dust may also contribute to inhibiting allergic responses and inflammatory responses which may be induced in the woodworkers; this is evident by the high level of Th1 and Treg cytokines which are known to antagonize inflammatory and allergic responses and the nonsignificant positive correlation of inflammatory cytokines and age of woodworkers.

## Figures and Tables

**Table 1:** Comparison of inflammatory cells counts between study populations.

Blood cells ×10^3^	Woodworkers mean (SD)	Nonexposed workers mean (SD)	*p* value
Total WBC's	5.13 ± 1.351	4.66 ± 1.648	0.05
Lymphocytes	2.331 ± 0.631	2.321 ± 0.770	0.902
Neutrophils	2.228 ± 0.664	1.719 ± 1.052	<0.001^∗∗∗^
Platelets	203.39 ± 50.584	156.78 ± 60.498	<0.001^∗∗∗^

*p* values: ^∗^*p* < 0.05, ^∗∗^*p* < 0.01, ^∗∗∗^*p* < 0.001; SD: standard deviation.

**Table 2:** Result of compared cytokine levels between woodworkers and nonexposed workers.

Cytokine	Woodworker (mean ± SD)	Nonexposed worker (mean ± SD)	Mean difference	95% CI	*T*	*p* value
*IL-1β*	10.26 (±2.59)	8.86 (±2.68)	1.40	0.60–2.19	3.47	0.0006^∗∗∗^
*IL-2*	21.68 (±5.74)	20.17 (±5.41)	1.51	-0.15–3.17	1.79	0.0758
*IL-4*	27.86 (±6.27)	19.55 (±5.52)	8.31	6.54–10.07	9.29	<0.001^∗∗∗^
*IL-6*	9.34 (±3.46)	7.32 (±4.59)	2.01	0.78–3.24	3.24	0.0015^∗∗^
*IL-10*	22.40 (±6.17)	18.63 (±6.80)	3.77	1.82–5.71	3.83	0.0002^∗∗∗^
*IL-12p70*	18.29 (±4.53)	13.12 (±3.78)	5.17	3.93–6.42	8.22	<0.001^∗∗∗^
*IL-13*	7.17 (±1.77)	6.25 (±1.76)	0.93	0.40–1.45	3.47	0.0007^∗∗∗^
*IL-17A*	13.30 (±2.66)	10.99 (±2.71)	2.30	1.50–3.10	5.68	<0.001^∗∗∗^
*IFN-γ*	18.63 (±4.39)	13.18 (±5.28)	5.45	3.99–6.91	7.39	<0.001^∗∗∗^

*p* values in parentheses; ^∗^*p* < 0.05, ^∗∗^*p* < 0.01, ^∗∗∗^*p* < 0.001; SD: standard deviation.

**Table 3:** Correlation between cytokines and ages of woodworkers.

Cytokines	Age	(*p* value)
IL-6	0.019	(0.8060)
IL-10	-0.022	(0.7768)
IL-1*β*	0.065	(0.3976)
IFN-*γ*	0.115	(0.1310)
IL-4	0.085	(0.2629)
IL-17A	0.070	(0.3569)
IL-2	-0.011	(0.8894)
E-selectin	0.003	(0.9666)
IL-13	0.017	(0.8214)
GCSF	0.068	(0.3750)
IL-12p70	0.135	(0.0747)
CD40L	-0.019	(0.8060)
Eotaxin	0.063	(0.4118)

*p* values in parentheses; ^∗^*p* < 0.05, ^∗∗^*p* < 0.01, ^∗∗∗^*p* < 0.001. Values were based on Spearman rank correlation.

## Data Availability

The datasets used to support the findings of this study are available from the corresponding author upon request.
